# Global hotspots and prospects of perimenopausal depression: A bibliometric analysis *via* CiteSpace

**DOI:** 10.3389/fpsyt.2022.968629

**Published:** 2022-09-10

**Authors:** Mingzhou Gao, Hao Zhang, Zhan Gao, Ya Sun, Jieqiong Wang, Fengqin Wei, Dongmei Gao

**Affiliations:** ^1^Innovation Research Institute of Traditional Chinese Medicine, Shandong University of Traditional Chinese Medicine, Jinan, China; ^2^Experimental Center, Shandong University of Traditional Chinese Medicine, Jinan, China; ^3^College of Traditional Chinese Medicine, Shandong University of Traditional Chinese Medicine, Jinan, China; ^4^Office of Academic Research, Shandong University of Traditional Chinese Medicine, Jinan, China

**Keywords:** perimenopausal depression, web of science, trends, knowledge domain, CiteSpace front psychiatry

## Abstract

**Background:**

Perimenopausal depression (PMD) is characterized by affective symptoms as well as menopause-specific somatic complaints and has attracted increasing attention over the past few decades. Using a bibliometric tool, this study aims to evaluate the origin, current hotspots, and research trends on PMD.

**Methods:**

Articles with research on PMD were retrieved from Web of Science Core Collection (WoSCC). We used the bibliometric method to analyze publication years, journals, countries, institutions, authors, research hotspots, and trends. We plotted the reference co-citation network and used keywords to analyze the research hotspots and trends.

**Results:**

A total of 209 publications related to PMD were identified from WoSCC on May 8, 2022. The number of publications concerning PMD every year shows an upward trend. Further analysis indicated that 209 articles were contributed by 45 countries, 288 institutions, and 501 authors. The United States contributed the most significant number of publications, followed by China. Harvard University is the core institution of PMD research, and Cohen’s work has had an important impact on another research. The occurrence and pathological mechanisms of depression during the menopausal transition from the knowledge base of PMD. All of them belong to the category of gynecology and psychosis, which reflects the focus of the research topics. Major depression, postmenopausal women, symptoms like hot flashes, and prevalence and risk factors are research hotspots in the PMD field. The frontiers in PMD field that will impact future research are anxiety, meta-analysis, association, and Beck Depression Inventory-II (BDI-II).

**Conclusion:**

These findings provide us with the core countries, institutions, and authors in PMD research and point out the direction of attention in this field. The current research focuses on depression, postmenopausal women, hot flashes, and other symptoms, as well as the prevalence and risk factors. The frontiers will be anxiety, meta-analysis, related factors, and depression assessment in future research.

## Introduction

Perimenopause is a natural physiological event in women that involves premenopause, menopausal transition, and early post-menopause (1). Within the female life cycle, women demonstrate a higher prevalence of or are at risk of depressive symptoms (2–4). Published research indicates that the highest rate of depression in perimenopause was 25.99% in Shanghai, China (5), and the prevalence of depression in perimenopausal and postmenopausal women in India is 42.47% (6). Furthermore, perimenopausal depression (PMD) is one of the forms of reproductive depression in women related to hormonal changes (7, 8). Women with PMD reported significantly decreased quality of life (QOL), social support, adjustment, suicidal thoughts, and increased disability and compared with non-depressed perimenopausal women (9).

There has been growing research on depression among perimenopausal women in recent years. However, there is no bibliometric analysis *via* CiteSpace on global research trends and hotspots in this field. CreateSpace is scientific software that has been widely used to identify frontier areas of current research by extracting burst terms from identifiers of titles, abstracts, descriptors, and bibliographic records (10). CiteSpace’s recognition in the field of research has increased considerably in recent years. It is used widely in many research fields such as medicine, geology, and others (11–13) and has promoted the rapid development of relevant research (14).

This study aims to investigate the knowledge domain and gain insights into emerging trends and hot spots of PMD by constructing a visualization network *via* CiteSpace.

## Methods

### Data source and search strategy

Data were extracted from the Web of Science Core Collection (WoSCC) and downloaded one day on May 8, 2022. The search strategy was as follows: TITLE = (postmenopausal depression) or TITLE = (menopausal depression) or TITLE = (depression in midlife women) or TITLE = (depression) and TITLE = (“menopause” or “perimenopause”). The search was conducted from the beginning of the database collection to May 8, 2022.

### Inclusion criteria and exclusion criteria

Publications related to PMD were identified from WoSCC published up to 2022 (retrieval deadline: 2022.5.8). And we excluded conference presentations, meeting abstracts, book reviews, news items, and corrections.

### Data analysis

This study utilized CiteSpace V.5.8.R2 to visually analyze the knowledge map of countries, institutions, authors, journals, references, and keywords concerning relevant research (15, 16). The visualization knowledge network created by CiteSpace consisted of nodes and lines. The nodes in the network stood for items, such as authors, countries, institutions, and cited references, and lines between the nodes represented cooperation, co-occurrence, or co-citation relationships. The size of each node indicated the count. Each node was represented by a series of citation rings representing different years, and the thickness of the ring was proportional to the citation count in the corresponding time zone. Purple rings indicate that these countries/regions, institutes, or authors have greater centrality indicating hot spots or pivotal points in a field (10–17).

## Results

### The trend of publication outputs

**FIGURE 1:**
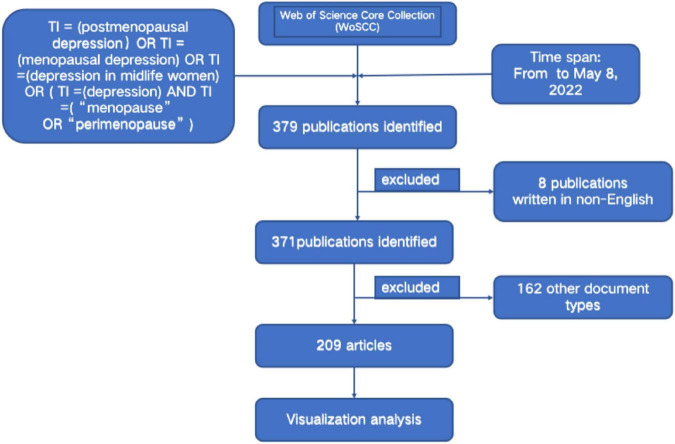
Flowchart of literature selection.

**FIGURE 2:**
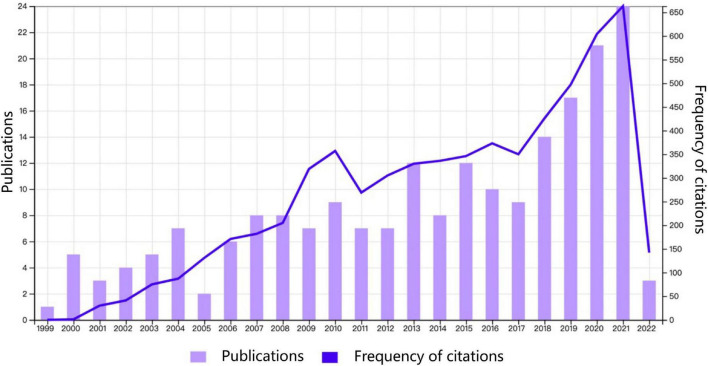
Trends of perimenopausal depression publications from 1999 to 2022.

A total of 379 publications related to PMD were identified, but 209 were finally included for further analysis (Figure 1). The number of publications and citations published in each period reflects the development trend of research in this field. As revealed in Figure 2, the number of publications concerning PMD has revealed an upward trend from 1999 to 2022, but the volatility is prominent. From 1999 to 2004, the steady increase in PMD research publications indicates that research in this field attracts increasing attention. In 2005, the study was at a standstill. After 2005, PMD field began to receive attention again, and the number of published articles increased considerably from 2017 to 2021, with publication outputs reaching 24 in 2022. Besides, the citation frequency of the report also shows that research in PMD field garnered incredible attention. The health problems of perimenopausal women have attracted attention.

### Quantitative and cooperation analysis

#### Countries/regions

**FIGURE 3:**
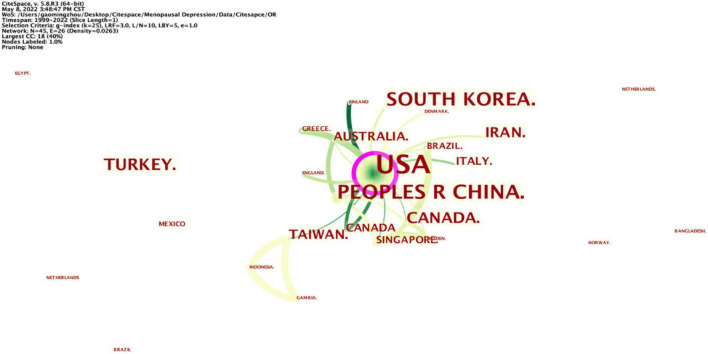
Distribution of publications from different countries/regions.

A total of 209 articles were published in 45 different countries. As Table 1 displays, the most significant number of publications came from the United States (79, 37.80%), China (25, 11.96%), South Korea (19, 9.09%), Turkey (13, 6.22%), and Canada (9, 4.31%), all of which published more than ten articles, except for Canada. The United States has the highest centrality (0.26), presented in Figure 3 with a purple circle. The lines between the processes denote cooperation between countries, and the wider the bars, the closer the cooperation. All of these indicate that the United States plays a leading role in the research in this field, and China still has a long way to go if it wants to catch up with this rate and even surpass it.

**TABLE 1:** Top 10 countries/regions related to PMD.

Rank	Count	Centrality	Countries
1	79	0.26	United States
2	25	0	Peoples R China.
3	19	0	South Korea.
4	13	0	Turkey.
5	10	0	Canada.
6	9	0	Iran.
7	8	0.06	Taiwan.
8	8	0	Poland.
9	6	0	Japan.
10	6	0	Australia.

#### Institutes

**FIGURE 4:**
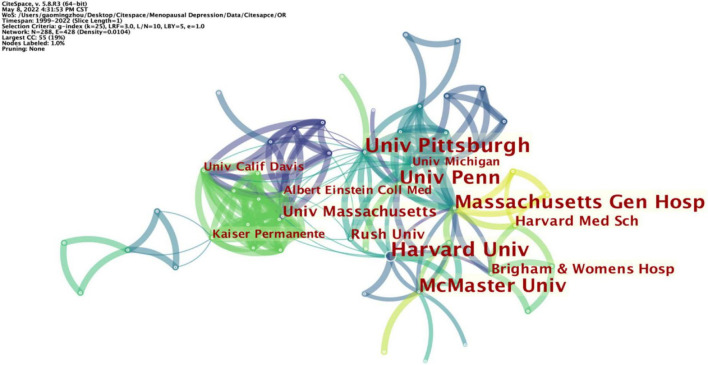
Distribution of publications from different institutions.

A total of 209 articles were published from 288 different institutions. Among the top ten institutions, Harvard University and the University of Pittsburgh have posted the same number of articles in this field, with ten published articles each. Notably, Harvard University is also the organization with the highest centrality, with a centrality of 0.02 (Table 2). In addition, Figure 4 indicates that inter-agency cooperation is relatively close and conducive to the continuity of inter-agency cooperative research.

**TABLE 2:** Top 10 institutions related to PMD.

Rank	Count	Institutions	Centrality	Institutions
1	10	Harvard Univ	0.02	Harvard Univ
2	10	Univ Pittsburgh	0.01	Univ Pittsburgh
3	9	McMaster Univ	0.01	McMaster Univ
4	9	Massachusetts Gen Hosp	0.01	Massachusetts Gen Hosp
5	8	Univ Penn	0.01	Univ Penn
6	5	Univ Massachusetts	0.01	Univ Massachusetts
7	4	Rush Univ	0.01	Univ Michigan
8	4	Poznan Univ Med Sci	0.01	Univ Calif Davis
9	4	Harvard Med Sch	0.01	Kaiser permanente
10	4	Brigham and Womens Hosp	0.01	Albert Einstein Coll Med

#### Authors and co-cited authors

**FIGURE 5:**
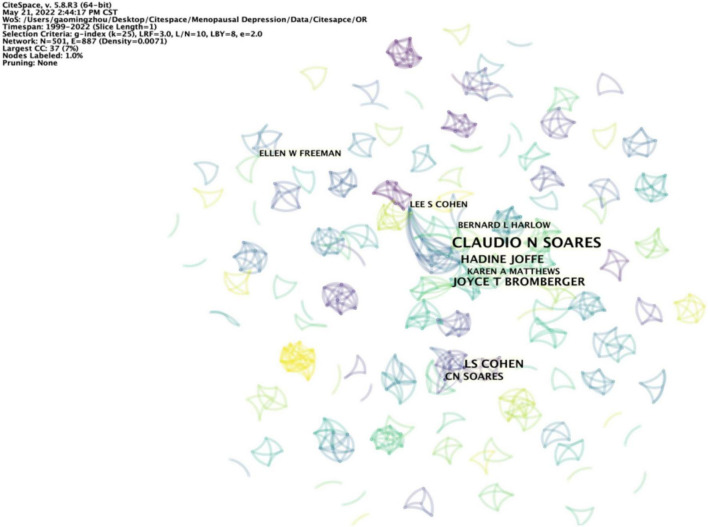
Visualization map of authors of publications about PMD.

A total of 501 authors were involved in publishing literature on PMD. As presented in Table 3, Soares had the highest number of published papers (13). Among the top 10 authors, we can observe that Cohen (0.01) has high centralities, showing that Cohen strongly influences other work (Table 3). Simultaneously, we found that 209 authors have not yet formed an extensive cooperation network, and scientific research cooperation is relatively scattered (Figure 5).

**TABLE 3:** Top 10 authors related to PMD.

Rank	Authors	Count	Centrality
1	Soares CN	13	0
2	Cohen LS	9	0.01
3	Bromberger JT	8	0
4	Joffe H	7	0
5	Freeman EW	5	0
6	Harlow BL	5	0
7	Kravitz HM	4	0
8	Matthews KA	4	0
9	Otto MW	4	0
10	Sammel MD	4	0

Co-cited authors are two or more authors cited by one or more papers simultaneously, and these authors constitute a co-cited relationship. Among the 612 co-cited authors, Bromberger (25) was the most frequently cited author, followed by Freeman (25) and Cohen (21) (Table 4). We noticed that Freeman and Cohen are leaders in this field, and their scientific research achievements play a guiding role in developing this field.

**TABLE 4:** Top 10 co-cited authors related to PMD.

Rank	Count	Co-cited authors	Centrality	Co-cited authors
1	27	Bromberger JT	0.32	Freeman EW
2	25	Freeman EW	0.26	Cohen LS
3	21	Cohen LS	0.26	American psychiatric association
4	13	Kessler RC	0.22	Amore M
5	13	Schmidt PJ	0.21	Avis Nancy E
6	11	Soares CN	0.17	Beck AT
7	10	Dennerstein L	0.16	Bromberger JT
8	10	Joffe H	0.13	Kessler RC
9	9	Beck AT	0.12	Schmidt PJ
10	7	Avis NE	0.12	Kendler KS

### Research topic analysis

#### Reference co-citation

**FIGURE 6:**
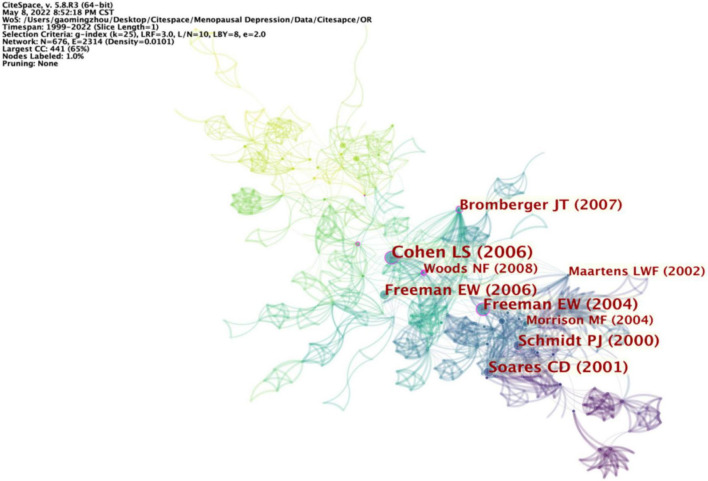
Visualization map of references involved in PMD.

Among 476 co-cited references retrieved, Table 5 and Figure 6 displays the 10 most frequently cited references; “Risk for new onset of depression during the menopausal transition: the Harvard study of moods and cycles” contributed by Cohen is the most frequently cited (23). In this article, Cohen explained that the transition to menopause increases the risk of depressive symptoms, and the article aroused great interest in follow-up research. Relevant directions have become research hotspots in terms of citation frequency.

**TABLE 5:** Top 10 co-cited references related to PMD in counts.

Rank	Count	Years	Co-cited references
1	23	2006	Cohen LS, 2006, ARCH GEN PSYCHIAT, V63, P385, DOI 10.1001/archpsyc.63.4.385
2	18	2004	Freeman EW, 2004, ARCH GEN PSYCHIAT, V61, P62, DOI 10.1001/archpsyc.61.1.62
3	17	2001	Soares CD, 2001, ARCH GEN PSYCHIAT, V58, P529
4	16	2000	Schmidt PJ, 2000, AM J OBSTET GYNECOL, V183, P414, DOI 10.1067/mob.2000.106004
5	16	2006	Freeman EW, 2006, ARCH GEN PSYCHIAT, V63, P375, DOI 10.1001/archpsyc.63.4.375
6	14	2007	Bromberger JT, 2007, J AFFECT DISORDERS, V103, P267, DOI 10.1016/j.jad.2007.01.034
7	10	2008	Woods NF, 2008, MENOPAUSE, V15, P223, DOI 10.1097/gme.0b013e3181450fc2
8	9	2002	Maartens LWF, 2002, MATURITAS, V42, P195, DOI 10.1016/S0378-5122(02)00038-5
9	9	2004	Morrison MF, 2004, BIOL PSYCHIAT, V55, P406, DOI 10.1016/j.biopsych.2003.08.011
10	8	2011	Bromberger JT, 2011, PSYCHOL MED, V41, P1879, DOI 10.1017/S003329171100016X

Besides, as depicted in Table 6, “Depressed mood during the menopausal transition and early post-menopause: Observations from the Seattle Midlife Women’s Health Study” contributed by Woods in 2008 has the highest centrality (0.22), which indicates that Woods’s findings have become a research hotspot.

**TABLE 6:** Top 10 co-cited references related to PMD in Centrality.

Rank	Centrality	Years	Co-cited references
1	0.22	2008	Woods NF, 2008, MENOPAUSE, V15, P223, DOI 10.1097/gme.0b013e3181450fc2
2	0.19	2004	Freeman EW, 2004, ARCH GEN PSYCHIAT, V61, P62, DOI 10.1001/archpsyc.61.1.62
3	0.18	2011	Bromberger JT, 2011, PSYCHOL MED, V41, P1879, DOI 10.1017/S003329171100016X
4	0.14	2016	Almeida OP, 2016, MENOPAUSE, V23, P669, DOI 10.1097/GME.0000000000000598
5	0.11	2006	Cohen LS, 2006, ARCH GEN PSYCHIAT, V63, P385, DOI 10.1001/archpsyc.63.4.385
6	0.1	2007	Bromberger JT, 2007, J AFFECT DISORDERS, V103, P267, DOI 10.1016/j.jad.2007.01.034
7	0.08	2010	Bromberger JT, 2010, ARCH GEN PSYCHIAT, V67, P598, DOI 10.1001/archgenpsychiatry.2010.55
8	0.08	2013	AmericanPsychiatricAssociationDSM-5TaskForce, 2013, DIAGNOSTIC STAT MANU, V5th, P0, DOI 10.1176/APPI.BOOKS.9780890425596
9	0.07	2002	Maartens LWF, 2002, MATURITAS, V42, P195, DOI 10.1016/S0378-5122(02)00038-5
10	0.07	1998	Burt VK, 1998, HARVARD REV PSYCHIAT, V6, P121, DOI 10.3109/10673229809000320

#### Journal co-citation

A total of 209 related to PMD were published in 124 academic journals. *Menopause: The Journal of the North American Menopause Society* (21) had the highest number of outputs, followed by Maturitas (11). Among the top 10 journals related to PMD, the *Journal of Affective Disorders* has the highest impact factor, 4.836. The JCR division of *Maturitas*, the *Journal of Affective Disorders*, and the *Journal of Clinical Psychiatry* are very high, Q1 (Table 7).

**TABLE 7:** Top 10 journals related to PMD.

Rank	Journals	Count	IF	JCR
1	Menopause the journal of the north American menopause society	21	2.953	Q2
2	Maturitas	11	4.342	Q1
3	Climacteric	8	3.005	Q2
4	Journal of affective disorders	8	4.839	Q1
5	Archives of womens mental health	6	3.633	Q2
6	Journal of clinical psychiatry	6	4.384	Q1
7	Archives of general psychiatry	5	–	–
8	Plos one	4	3.24	Q2
9	BMC womens health	3	2.809	Q2
10	Journal of clinical psychopharmacology	3	3.153	Q3

**FIGURE 7:**
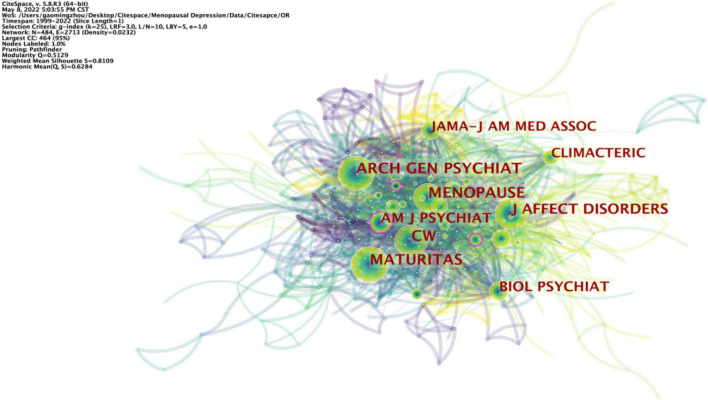
Visualization map of journals on PMD.

Among the top 10 co-cited academic journals, five journals have been cited a hundred times more than the other 474 journals. As shown in Table 8 and Figure 7, the journals with the highest number of citations are the *Archives of General Psychiatry* (133), followed by *Maturitas* (127). Besides, the journals with the highest centrality are *Am J Obstet Gynecol* (0.18), followed by *Acta Psychiat Scand (0.15).*

**TABLE 8:** Top 10 co-cited journals related to PMD.

Rank	Count	Cited journals	Centrality	Cited journals
1	133	Arch Gen Psychiat	0.18	Am J Obstet Gynecol
2	127	Maturitas	0.15	Acta Psychiat Scand
3	119	Menopause	0.14	Am J Epidemiol
4	112	CW	0.11	Brit J Psychiat
5	109	J Affect Disorders	0.1	Am J Psychiat
6	82	Am J Psychiat	0.1	Arch Intern Med
7	75	Biol Psychiat	0.1	Applied Psychological Measurement
8	67	Climacteric	0.09	Biol Psychiat
9	66	JAMA-J Am Med Assoc	0.09	Ann Intern Med
10	58	J Clin Psychiat	0.07	Am J Public Health

**FIGURE 8:**
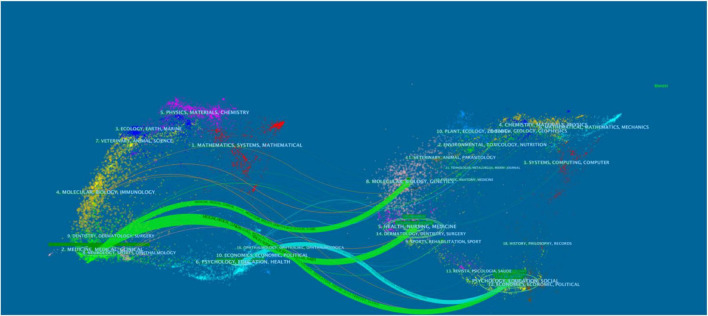
Dual-map overlay of journals on PMD.

The dual-map overlay of journals demonstrates the relationship distribution among journals, citing journals on the left and the right. The colored paths between them suggest the aforementioned relationships. A green path in Figure 8 indicates that documents published in molecular/biology/genetics, health/nursing/medicine, and psychology/education/social journals are often cited by medicine/medical/clinical and neurology/sports/ophthalmology journals. A green path in Figure 8 indicates that psychology/education/health journals often cite the documents published in economics/economic/political journals.

### Research hotspots and frontiers analysis

#### Keywords co-occurrence

**FIGURE 9:**
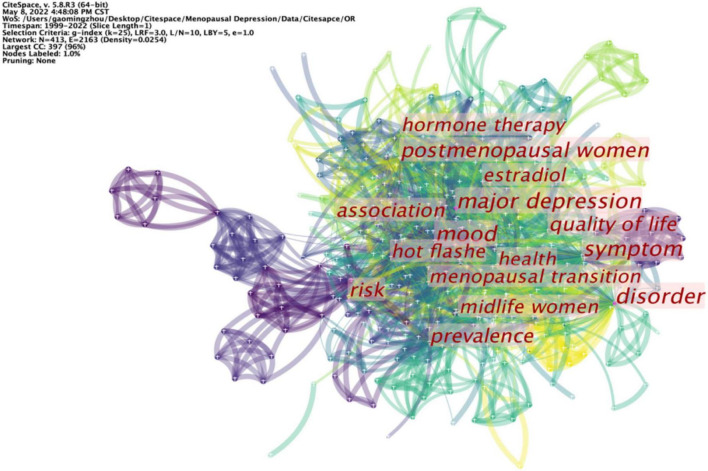
Co-occurring keywords map.

High-frequency keywords represent a hot topic in a research field, while high-centrality keywords reflect the position and influence of the corresponding research content in this research field. As revealed in Table 9, hot keywords in the frequency order were symptom (68), mood (33), postmenopausal women (28), major depression (26), and disorder (23). Hot keywords in the centrality order comprised major depression (0.26), disorder (0.21), mood (0.19), postmenopausal women (0.17), and symptom (0.14). Other keywords included hot flashes, risk, prevalence, quality of life, and so on (Figure 9).

**TABLE 9:** Top 10 keywords in terms of frequency and centrality on PMD.

Rank	Count	Keywords	Centrality	Keywords
1	68	Symptom	0.26	Major depression
2	33	Mood	0.21	Disorder
3	28	Postmenopausal women	0.19	Mood
4	26	Major depression	0.17	Postmenopausal women
5	23	Disorder	0.14	Symptom
6	22	Hot flashe	0.14	Quality of life
7	22	Risk	0.13	Prevalence
8	21	Prevalence	0.12	Midlife women
9	20	Quality of life	0.11	Age
10	18	Health	0.09	Risk

#### Emerging trends

**FIGURE 10:**
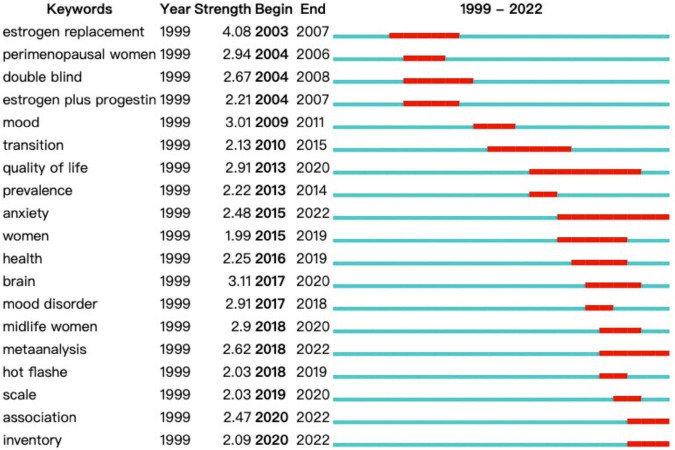
Keywords with the most robust citation bursts in published articles on PMD.

Figure 10 displays the top 19 keywords with the strongest citation bursts in published articles on PMD. The blue line represents the time interval, and the red line refers to the duration of the citation burst. In the keyword’s citation burst detection analysis, Estrogen replacement was the strongest burst keyword in 2003, with a burst strength of 4.08, followed by mood (3.01). Five frontiers in PMD field that will impact future research are anxiety, meta-analysis, association, and inventory.

## Discussion

### General information

The harm of PMD to women is gradually attracting attention. Using PMD as a keyword in this study, we searched relevant studies included in WOS until May 8, 2022, and finally included 209 studies. PMD research began in 1999, “Estrogen therapy for depression in postmenopausal women” is the work that started in this period. After 1999, PMD research gradually aroused researchers’ interest and showed an upward trend, reaching a peak in 2021. Further analysis indicated that 209 articles were contributed by 45 countries, 288 institutions, and 501 authors. Furthermore, the United States contributed the most significant number of publications, followed by China. The United States is in the core leadership position regarding the number of published articles and centrality. The significant development made by China is also full of potential for future research. As for contributing intuitions, Harvard University is the core institution of PMD research, and its research direction and findings play an important guiding role in PMD research. Besides, close scientific research cooperation has not been formed in PMD research between authors, and Cohen’s work has an important impact on another research.

### Research fundamentals

The top co-cited articles are often considered fundamental and a basis for a specific research field. In this study, “Risk for new onset of depression during the menopausal transition: the Harvard study of moods and cycles” contributed by Cohen ranked first in the frequency of citation, and “Depressed mood during the menopausal transition and early post-menopause: Observations from the Seattle Midlife Women’s Health Study” contributed by Woods in 2008 ranked first in centrality. The menopause transition is a disruptive process that causes symptoms such as mood disruption in most women (18). Thus, clinicians must recognize depressive symptoms of the transition and be prepared to offer treatment to mitigate these symptoms (19). The occurrence and pathological mechanisms of depression during the menopausal transition from the knowledge base of PMD.

### Research topic

The highly cited journals reflect the research topic in PMD field to a certain extent. This study found that *Menopause: The Journal of the North American Menopause Society* had the highest number of outputs. Besides, the *Archives of General Psychiatry* has the highest number of citations, and *Am J Obstet Gynecol* has the highest centrality. All of them belong to the category of gynecology and psychosis, which reflects the focus of the research topics. Furthermore, the dual-map overlay of journals demonstrates the relationship distribution among journals such as molecular/biology/genetics, health/nursing/medicine, psychology/education/social, etc.

### Research hotspots and frontiers

Keywords are the research themes and core contents of the literature, suggesting research hotspots and frontiers in a particular field. The four research hotspots in the field of PMD are as follows:

#### Major depression

Depression is a common mood disorder with a wide array of symptoms affecting somatic, cognitive, affective, and social processes in adolescence, midlife, and the elderly (20–22). Among midlife women, depression is one of the leading causes of disease-related disability. They are nearly twice as likely as men to suffer from an episode of depression (22). Besides, evidence generally suggests that most midlife women who experience a major depressive episode during perimenopause have experienced a prior episode of depression. Midlife depression presents with classic depressive symptoms, commonly combined with menopause symptoms (23).

#### Postmenopausal women

Menopause, defined by amenorrhea for 12 consecutive months, is determined retrospectively and represents a permanent end to menses. Many physical changes occur during the menopausal transition and beyond (24). Established studies have indicated that minor depression predominates in perimenopausal women (21.4%), and major depression predominates in postmenopausal women (59.3%) (25). Besides, postmenopausal women also have an increased risk of other diseases, such as dyspareunia (26), hypertension (27), and others.

#### Related symptoms like hot flashes

Hot flashes (HFs) are a sensation of heat that can be accompanied by facial flushing, perspiration, chills, heart palpitations, night sweats, and anxiety (28). For women with perimenopausal syndrome, hot flashes are its characteristic manifestations (29, 30). Studies have indicated that HF disturbs sleep, causing insomnia and increasing vulnerability to depression (31–33). Besides, hot flashes are a common stressful symptom for individuals with cancer and other diseases (34). Therefore, studies in pathophysiology and treatment of hot flashes are becoming prevalent (35–37).

#### Prevalence and risk factors

To understand the development trends of a disease, it is essential to master its incidence rate and risk factors. For instance, the prevalence of major depression among middle-aged women was 26.09% in the rural area of Kerala (38). The highest rate of depression among perimenopausal women was 25.99% in Shanghai, China (5). Relevant studies are still being performed (6). Besides, managing the risk factors of PMD remains a hotspot for improving the health of perimenopausal women (39, 40).

The four frontiers in PMD field that will impact future research include anxiety, meta-analysis, association, and inventory.

#### Anxiety disorder

Anxiety is the feeling of fear that occurs when faced with threatening or stressful situations (41). About 85% of patients with depression have significant anxiety (42). Established studies reveal that anxiety increases during the menopausal transition with depressed mood (43–45). Furthermore, anti-anxiety research will become a research frontier in PMD field (46–48).

#### Meta-analysis

A meta-analysis is a statistical method for combining the results of different studies on the same topic. It may resolve conflicts among studies and plays a vital role in medical research (49, 50). Meta-analysis is being used in research on perimenopausal women and has made important discoveries. For instance, Yadav et al. found that depression prevalence in perimenopausal and postmenopausal women in India is 42.47% (6). By meta-analysis, long-term hormone therapy for perimenopausal and postmenopausal women is also vital for evaluating the effectiveness and safety of treatment methods (51). More meta-analyses of PMD will be conducted in the future.

#### Association study

Since PMD is not a single disease but is very complex, association studies are being carried out. In terms of mechanisms, perimenopause is associated with reproductive and hormonal changes (1). Thus, perimenopausal mood disorders are related to other reproductive-related conditions such as premenstrual syndrome (52, 53). Besides, research on the association between low bone mineral density and periodontitis in perimenopausal women (54), the association between homocysteine, C-reactive protein, lipid levels, and sleep quality in perimenopausal and postmenopausal women (55), and so on are frontiers in subsequent research.

#### Beck Depression Inventory-II application

The Beck Depression Inventory-II (BDI-II) is viewed as a cost-effective questionnaire for measuring the severity of depression and is frequently used (56–58). As a relevant psychometric instrument, BDI-II shows high reliability and can discriminate between depressed and non-depressed subjects for research and clinical practice worldwide (58, 59). Therefore, using BDI-II to measure depression in perimenopausal women is well recognized, and the tool will be widely used in the future (60).

## Strengths and limitations

This is the first study to use CiteSpace to perform bibliometric analysis and provide a visual display of publications on PMD from the cooperation among authors, countries, and institutions to hot spots. However, our study still has some limitations. Since the study was limited to CiteSpace software, we analyzed only English studies in WOS; therefore, the data may be insufficient. Our results may be inapplicable to research published in other languages. Furthermore, Citespace’s analysis cannot give a direct answer for clinicians who are looking for effective interventions or researchers who are looking for novel research methods, or clinical teachers who are looking for up-to-date information about a topic. Indirectly, however, it points clinicians, teachers, and investigators to sources that can answer their questions.

## Conclusion

A current study *via* CiteSpace suggested United States, Harvard University, and Cohen LS are the core of PMD research from countries, institutions, and authors. Major depression, postmenopausal women, symptoms like hot flashes, and prevalence and risk factors are research hotspots in PMD. Four frontiers in the area of PMD that impact research are anxiety symptoms, meta-analysis research, association and difference, and BDI-II application in the future.

## Author contributions

MG designed the study, wrote, and revised the draft manuscript. MG, HZ, ZG, YS, and JW performed the literature search, retrieval, and data collection. MG conducted data visualization and graphical interpretation with HZ. DG and FW provided critical assistance and funding. All authors contributed to and approved the final draft of the manuscript before submission.
